# Supplementary material for: Chemical Content of Five Molluscan Bivalve Species Collected from South Korea: Multivariate Study and Safety Evaluation
Source: Foods. 2021 Nov 3;10(11):2690. doi: 10.3390/foods10112690 (PMC8623076; doi:10.3390/foods10112690)

Chemical Content of Five Molluscan Bivalve Species Collected from South Korea: Multivariate Study and Safety Evaluation

Jelena Mutić[1,2], Vesna Jovanović[1,2], Liesbeth Jacxsens[3], Jannes Tondeleir[3], Petar Ristivojević[1,2], Sladjana Djurdjić[1], Andreja Rajković[3], Tanja Ćirković Veličković[1,2,3,4,*]

[1]*University of Belgrade - Faculty of Chemistry, Studentski trg 12-16, 11000 Belgrade, Serbia*

[2]*Ghent University Global Campus, Department of Molecular Biotechnology, Environmental Technology and Food Technology, 21985Incheon, South Korea*

[3] *Ghent University, Department of Food Technology, Safety and Health, Faculty of BioScience Engineering, B-9000 Ghent, Belgium*

[4]*Serbian Academy of Sciences and Arts, Department of Chemical and Biological Sciences, 11000Belgrade, Serbia*

Supplementary material

Content

Table S1. Taxonomic ranks, common name and number of samplesof bivalve species

Table S2. Water content in bivalve samples (VP- *Venerupis.philippinarum*; AB- *A.broughtonii*; TG- *T.granosa*; MY- *M.yessoensis*; AI- *A.irradians*)

Table S3. Instrument operating conditions for determination of elements

Table S4. Results of determination of elements in certifiedreference material DORM-2(μg/g, dry weight)

Table S5. Statistical performances of the PLS-DA models

Table S6. The calculated content of essential (mg) and other (μg) elements in one portion size of five bivalve species. For the calculation of element intake, the 25-g of wet mass of bivalve was used as a portion size.

Table S7. Results of the consumption survey on frequency of consumption for Yesso scallop and recalculation towards daily consumption to construct a discrete distribution of consumption frequency.

Figure. S1. Distribution of chronic consumption of Yesso scallop (*Mizuhopectenyessoensis*) by the Korean population (expressed g/day per person). Data was generated via @Risk software.

Figure. S2. Example of chronic probabilistic exposure to Cd (μg/kg BW per day) for Korean population by consumption ofYesso scallop (*Mizuhopectenyessoensis*). Data was generated via @Risk software and in UB scenario.

Table S1. Taxonomic ranks, common name and number of samplesof bivalve species

	Clams			Scallops	
No. of samples	n=15	n=7	n=12	n=8	n=6
Common name	Manila clam<br>Japanese carpet shell	Big blood clam<br>Blood cockle	Small blood clam<br>Blood cockle	Atlantic bay scallop<br>Bay scallop	Yesso scallop<br>Giant Ezo scallop
**Taxonomic rank**					
Phylum	Mollusca	Mollusca	Mollusca	Mollusca	Mollusca
Class	Bivalvia	Bivalvia	Bivalvia	Bivalvia	Bivalvia
Order	Venerida	Arcida	Arcida	Pectinida	Pectinida
Family	Veneridae	Arcidae	Arcidae	Pectinidae	Pectinidae
Genus	Venerupis	Anadara	Tegillarca	Argopecten	Mizuhopecten
Species	*Venerupis philippinarum*	*Anadara broughtonii*	*Tegillarca granosa*	*Argopecten irradians*	*Mizuhopecten yessoensis*

Table S2. Water content (%) in bivalve samples (VP- *Venerupis philippinarum*; AB- *Anadara broughtonii*; TG- *Tegillarca granosa*; MY- *Mizuhopecten yessoensis*; AI- *Argopecten irradians*)

Sample	Water (%)	Sample	Water (%)	Sample	Water (%)	Sample	Water (%)	Sample	Water (%)
VP 001	83.3	AB 001	84.5	TG 001	76.7	MY 001	83.7	AI 001	86.6
VP 002	86.8	AB 002	82.7	TG 002	77.3	MY 002	85.0	AI 002	91.5
VP 003	86.3	AB 003	82.9	TG 003	76.4	MY 003	84.7	AI 003	88.4
VP 004	87.4	AB 004	83.7	TG 004	79.0	MY 004	85.5	AI 004	86.8
VP 005	83.1	AB 005	83.3	TG 005	77.4	MY 005	86.7	AI 005	88.9
VP 006	86.4	AB 006	86.3	TG 006	81.3	MY 006	87.2	AI 006	88.5
VP 007	77.0	AB 007	87.0	TG 007	81.2			AI 007	90.5
VP 008	88.8			TG 008	80.1			AI 008	88.3
VP 009	79.1			TG 009	74.9				
VP 010	86.9			TG 010	83.9				
VP 011	88.1			TG 011	86.5				
VP 012	86.4			TG 012	86.4				
VP 013	87.8								
VP 014	84.4								
VP 015	85.9								

Table S3. Instrument operating conditions for determination of elements

ICP MS	Spectrometar	ICP Q MS
	Rf power (W)	1548
	Gas flows (L/min)	13.9: 1.09; 0.8
	Acquisition time	3 x 50 ms
	Points per peak	3
	Dwell time (ns)	10
	Detector mode	Pulse
	Replicates	3
	Measured isotope	52Cr, 55Mn, 59Co, 60Ni, 65Cu, 66Zn,75As, 82Se,111Cd, 202Hg, 208Pb
ICP OES	Spectrometar	iCAP 6500 Thermo scientific
	Nebulizer	Concentric
	Spray chamber	Cyclonic
	Radio frequency power (W)	1150
	Principal argon flow rate (L/min)	12
	Auxiliary argon flow rate (L/min)	0.5
	Nebulizer flow rate (L/min)	0.5
	Sample flow rate (ml/min)	1.0
	Detector	CID86
	Selected wavelenghts (nm)	Ca (373.6), Mg (279.5), Na (589.5), K (766.4) Fe (259.9)

Table S4. Results of determination of elements in certified reference material DORM-2(µg/g, dry weight)

Element	DORM-2 (dogfish muscle)		
	**Certified± uncertainty***	**Found ± uncertainty**	**Recovery (%)**
As	18.0±1.1	17.13 ± 0.05	95.17
Cd	0.043±0.008	0.036 ± 0.001	83.72
Co	0.182±0.031	0.169 ± 0.001	92.86
Cr	34.7±5.5	31.1 ± 0.3	89.74
Cu	2.34±0.16	2.40 ± 0.04	102.56
Fe	142 ± 10	144.7 ± 0.4	101.36
Mn	3.66±0.34	3.49 ± 0.04	95.36
Ni	19.4±3.1	16.6 ± 0.2	85.57
Pb	0.065±0.007	0.07±0.01	107.69
Se	1.40±0.09	1.43 ± 0.03	102.14
Zn	25.6±2.3	27.02 ± 0.18	105.55
Hg	4.640±0.260	4.560 ±0.460	98.28

*Uncertainty for 95 % confidence level (coverage factor k = 2)

Table S5. Statistical performances of the PLS-DA models

	Venerupis philippinarum	*Anadara broughtonii*	*Tegillarca granosa*	*Argopecten irradians*	*Mizuhopecten yessoensis*
R^2_{cal}	0.849162	0.405639	0.678967	0.871248	0.839146
R^2_{CV}	0.804761	0.132479	0.360329	0.83214	0.722855
RMSEC	0.180018	0.272098	0.245344	0.133725	0.13264
RMSECV	0.205216	0.350073	0.369418	0.152761	0.174608

Table S6. The calculated content of essential (mg) and other (μg) elements in one portion size of five bivalve species. For the calculation of element intake the 25-g of wet mass of bivalve was used as a portion size.

	Venerupis philippinarum	*Anandara broughtonii*	*Tegillarca granosa*	*Argopecten irradians*	*Mizohopecten yessoensis*
Essential elements (mg/25 g of one portion)					
Cu	0.019	0.027	0.030	0.019	0.035
Zn	0.209	0.294	0.312	1.062	0.363
Fe	0.875	1.395	1.715	0.350	0.385
Mn	0.033	0.090	0.093	0.63	0.014
Co	0.00375	0.00125	0.0015	0.00175	0.00075
Other elements (μg/25 g of one portion)					
Cr	2.0	2.0	2.5	1.5	1.75
Ni	14.5	2.0	3.0	1.0	18.5
Se	13	8.75	13.25	9.25	10.5

Table S7. Results of the consumption survey on frequency of consumption for Yesso scallop and recalculation towards daily consumption to construct a discrete distribution of consumption frequency.

Number of respondents	Percentage of respondents (%)	Consumption frequency	Recalculated daily consumption frequency
0	0	Daily	1
1	0.70	2-3 times a week	2.5/7
0	0	1 time a week	1/7
4	2.82	2-3 times a month	2.5/12
10	7.04	1 time a month	1/12
15	10.56	5-6 times a year	5.5/365
43	30.28	2-3 times a year	2.5/365
37	26.06	1 time a year	1/365
32	22.54	never	0

Figure. S1. Distribution of chronic consumption of Yesso scallop (*Mizuhopectenyessoensis*) by the Korean population (expressed g/day per person). Data was generated via @Risk software.

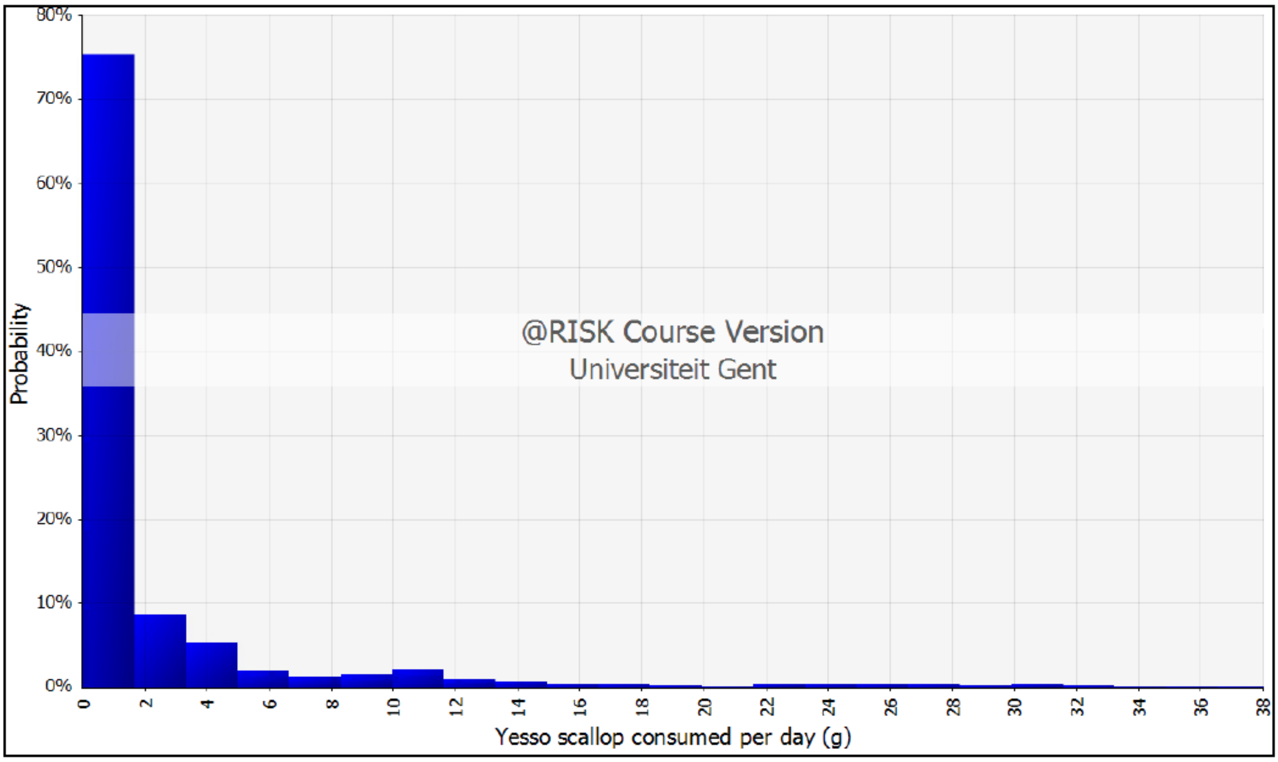

Figure. S2. Example of chronic probabilistic exposure to Cd (µg/kg BW per day) for Korean population by consumption ofYesso scallop (*Mizuhopectenyessoensis*). Data was generated via @Risk software and in UB scenario.

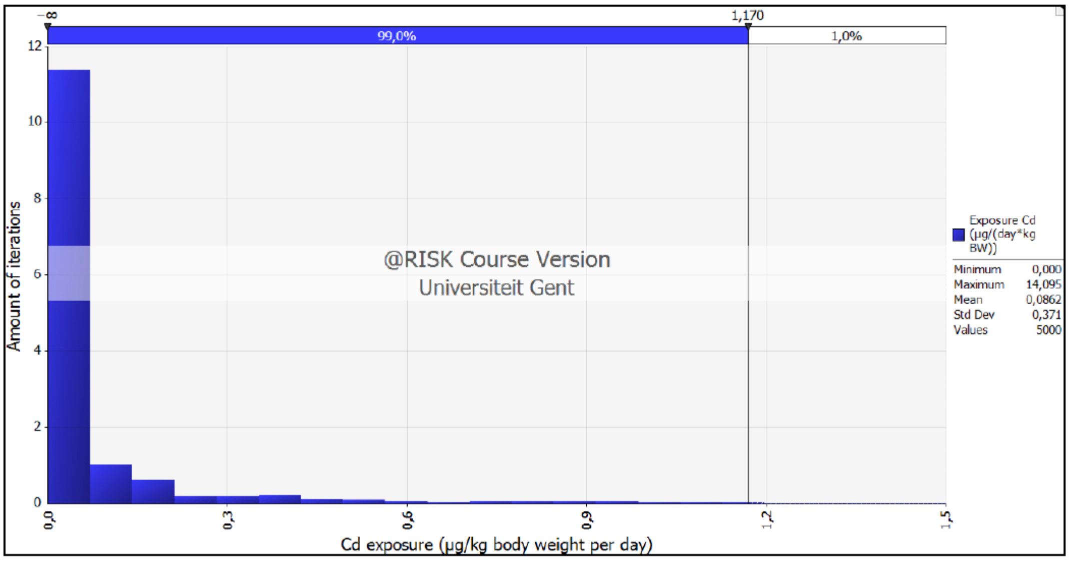

Supplement: Supplementary file 1 [file foods-10-02690-s001.zip › foods-1408644-supplementary.pdf]
